# Renewing the Federal Commitment to Advance Children’s Health: The President’s Task Force on Environmental Health Risks and Safety Risks to Children

**DOI:** 10.1289/ehp.1511016

**Published:** 2016-01-01

**Authors:** Ruth A. Etzel, Sandra N. Howard

**Affiliations:** 1 U.S. Environmental Protection Agency, Washington, DC USA; 2 U.S. Department of Health and Human Services, Washington, DC USA

Eighteen years ago President William Clinton issued Executive Order 13045 calling for each federal agency to “ensure that its policies, programs, activities, and standards address disproportionate risks to children that result from environmental health risks or safety risks” (Clinton and Gore 1997). As part of this Executive Order, Clinton established the President’s Task Force on Environmental Health Risks and Safety Risks to Children (U.S. EPA 2015a). Today the Task Force, which is cochaired by the U.S. Environmental Protection Agency (EPA) and the U.S. Department of Health and Human Services (HHS), continues its collaborative work and met recently to renew the federal commitment to advance children’s health.

The 14 October 2015 meeting was hosted by EPA Administrator Gina McCarthy and HHS Secretary Sylvia Mathews Burwell. Participants, who included representatives of 20 federal departments, agencies, and offices, reviewed the Task Force’s recent accomplishments and reiterated its important role in accomplishing the goals set out by the Executive Order:

identify priority risks and issues that can be addressed by interagency efforts;
develop strategies to protect children’s health;
recommend and implement interagency actions; and
communicate information to decision makers for use in protecting children’s environmental health and safety.

One of the major areas of interest for the Task Force is asthma disparities in children. In 2012 the Task Force issued a Coordinated Federal Action Plan to Reduce Racial and Ethnic Asthma Disparities, and its implementation is now well under way (U.S. EPA 2015b). The action plan calls for new policies, community programs, and research to reduce the burden of asthma among minority children and those with family incomes below or near the poverty level.

The plan leverages existing federal resources to address well-known preventable factors that contribute to asthma. For example, HHS, the EPA, and the Department of Housing and Urban Development (HUD) teamed up to encourage coverage of in-home asthma interventions by sponsoring regional asthma summits, which included meetings in Baltimore, Philadelphia, Kansas City, Denver, and Los Angeles. This engagement model brings together state and local entities across sectors to promote a collaborative approach in reducing asthma disparities.

Creation of healthy settings for children is another important focus of the Task Force. HUD is leading an effort involving several federal partners to implement recommendations in the report *Advancing Healthy Housing—A Strategy for Action*, which showcased initiatives to mitigate unsafe housing conditions and a shortage of safe, affordable housing for low-income families (Healthy Homes Workgroup 2013). A significant advance took place in mid-November 2015 when HUD Secretary Julián Castro announced a proposed rule that would require each public housing agency administering public housing to implement a smoke-free policy (HUD 2015). He was joined by the Surgeon General of the U.S. Public Health Service, Dr. Vivek Murthy, who has made promotion of tobacco-free living one of his highest priorities. More than 700,000 housing units, many with young children, would be affected by this rule (HUD 2015). The period for public comment ends 16 January 2016.

A relatively new workgroup within the Task Force is focusing attention on the impacts of climate change on children’s health. In 2014 the Task Force held a workshop on the emerging issue of the impacts of climate change on children’s health. The workshop helped inform every chapter of the U.S. Global Change Research Program’s forthcoming *Climate and Health Assessment* (U.S. GCRP 2015). The Task Force also solicited nongovernmental organizations, the public, and state, local, and federal governments to submit stories about actions they are taking to protect children’s health against the impacts of climate change (NIEHS 2015).

Among the inspiring stories collected so far is the May 2015 adoption of a resolution by the California Parent Teacher Association (PTA) Convention that officially declares climate change to be a children’s health issue (CA PTA 2015). The resolution calls for the education of parents on the impacts of climate change on children’s health and future welfare as well as the development of students’ own climate and energy literacy. By adopting this resolution, the state PTA put climate change on the agenda of local chapters throughout the state with the goal of mobilizing more than 800,000 California PTA members.

The Task Force member agencies, including the National Institute of Environmental Health Sciences, the Consumer Product Safety Commission, and the Centers for Disease Control and Prevention, have also worked together in assessing the impact of chemicals in the environment on children. A key achievement of the Task Force has been the identification of cross-agency biospecimen resources to support studies measuring children’s chemical exposures.

Because no single federal agency covers the wide array of issues that affect children’s environmental health, collaboration is essential. The Task Force has proven to be an excellent model for interagency collaboration to protect children’s health by providing a focus on the often overlooked contribution of environmental factors, be they chemical, biological, or social. As representatives of participating agencies reflected in October on their major achievements, they renewed their commitment to working together on important children’s health risks and safety risks. A new work plan to guide the group’s efforts over the next year and into the future is in development and is expected to be finalized in 2016.
